# Prehospital invasive vs. non-invasive blood pressure monitoring: Impact on shock index at hospital admission in critically ill patients – a prospective intervention study

**DOI:** 10.1186/s13049-025-01467-3

**Published:** 2025-10-15

**Authors:** Jakob Ule, Tobias Hüppe, Julian Thiel, Benedikt Merscher, David Conrad, Thomas Schlechtriemen, Thomas Volk, Ulrich Berwanger

**Affiliations:** 1 Department of Anesthesiology, Intensive Care and Pain Therapy, Saarland University Medical Center, Kirrberger Straße 100, 66421 Homburg (Saar), Germany; 2 Medical Director of Saarland Emergency Services and Fire Brigade Alerting (ZRF Saar), Saarpfalz-Park 9, 66450 Bexbach, Germany; 3 Department of Anesthesiology, Surgical Intensive Care and Pain Medicine, Marienhaus Clinic St. Elisabeth Saarlouis, Kapuzinerstraße 4, 66740 Saarlouis, Germany; 4 Saarland University, Anaesthesiology, 66421 Homburg, Germany

**Keywords:** Arterial line, Invasive blood pressure monitoring, Pre-hospital critical care, Intra-arterial blood pressure, Post-resuscitation care, Shock index

## Abstract

**Objectives:**

Hypotension and shock are potential modifiable contributors to adverse outcome. Inhospital, invasive blood pressure (IBP) monitoring is standard, while prehospital care mainly uses non-invasive blood pressure measurement. This study tested whether prehospital IBP monitoring improves shock index (SI) at hospital admission.

**Methods:**

This prospective interventional study included patients requiring prehospital intubation, catecholamines, or fluid resuscitation. Patients were assigned to prehospital IBP or Non-IBP group – according to the directives of the emergency physician. Primary endpoint was the SI at hospital admission. Secondary endpoints included catecholamines doses, fluid volume and arterial blood gas parameters (pH, lactate, base excess) at admission. Multiple regression analysis assessed whether IBP independently influenced SI at hospital admission.

**Results:**

392 patients were enrolled, and 19.6% (n = 77) had prehospital IBP. The IBP group had a significantly lower shock index at hospital admission (mean ± SD: 0.77 ± 0.4 with IBP vs. 0.93 ± 0.5 with NIBP; p = 0.002). Multiple regression analysis showed that IBP was independently associated with a lower shock index. IBP patients received more catecholamine boluses (2.1 ± 2.5 vs. 1.2 ± 1.8; p < 0.001), had more frequent use of continuous catecholamines (35.1% vs. 21.6%; p = 0.017), higher pH (7.34 ± 0.13 vs. 7.25 ± 0.16; p < 0.001) and less negative base excess (-3,8 ± 5.2 vs. -6.0 ± 7.8; p = 0.004) while lactate levels were lower (3.6 ± 3.2 vs. 4.4 ± 4.2; p = 0.047).

**Conclusions:**

Prehospital IBP monitoring significantly was associated with a decreased shock index at hospital admission in critically ill patients, likely due to earlier detection of hypotension and targeted hemodynamic therapy. IBP should be considered in patients receiving catecholamines.

**Graphical Abstract:**

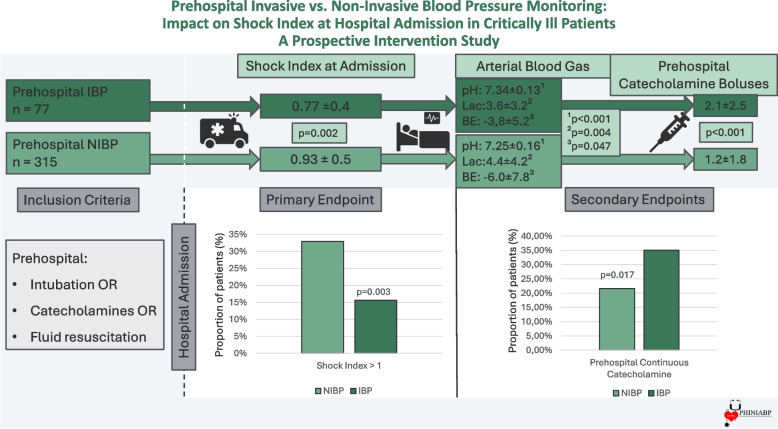

**Supplementary Information:**

The online version contains supplementary material available at 10.1186/s13049-025-01467-3.

## Background

Hypotension and shock represent significant, potentially modifiable contributors to prolonged organ dysfunction and increased mortality [1, 2]. Among various surrogate markers of tissue hypoperfusion, the shock index stands out as the most reliable predictor of mortality risk in these emergency patients [3]. A shock index greater than 1 is an independent risk factor of mortality and is not influenced by comorbidities or medications [4]. Optimal therapy necessitates prompt identification and close hemodynamic monitoring. Invasive blood pressure (IBP) is the gold standard for immediate detection of alterations in blood-pressure and therapy [5, 6]. While IBP monitoring is standard practice for critically ill patients in the hospitals, most emergency medical systems continue to rely on noninvasive blood pressure (NIBP) monitoring—despite proven feasibility of prehospital IBP placement [7–9]. Moreover, NIBP readings can be highly inaccurate [10], especially in hypotensive emergency situations [11, 12], and inaccuracy increases when multiple inotropes are administered [13]. We hypothesize that early implementation of IBP monitoring in the prehospital setting in critical ill patients is associated with improved hemodynamic stability, as measured by the shock index at the time of hospital admission.

## Method

### Ethics

This study was a part of the PHINIABP (PreHospital Invasive vs. Non-Invasive Blood Pressure) study and was registered with German Clinical Trials (ID DRKS00030477) and approved by the regional ethics committee (Ärztekammer Saarland, Saarbrücken, Germany, Identification Number 158/22, September 13, 2022). Written informed consent was obtained from patients or their legal representatives. This manuscript adheres to the TREND statement checklist- in Supplement 1.

### Study design

This study was a prospective interventional nonrandomized trial. The primary endpoint was the shock index at hospital admission, with the hypothesis that patients in the IBP group have a significantly lower shock index compared with those with NIBP. Secondary endpoints were the number of prehospital administrations of catecholamines in any dose (excluding epinephrin for cardiopulmonary resuscitation and intramuscular administration), rate of continuous catecholamine therapy, volume of fluid administration and the values from the first arterial blood gas analysis in the hospital: pH, lactate level and base excess. Patients with missing data were excluded. The study power was set at 0.8 with a significance level of α 0.05. The study started with an initial pilot phase involving 50 patients in the intervention group, followed by a power analysis. In the conditional power analysis conducted on 29.05.2024, a multiple regression model including two significant confounders with a combined R^2^ of 0.139 was used. Out of the aforementioned population, the calculation took the distribution difference into account, using a total of 253 patients, including 50 in the intervention group. The effect of IBP was additional R^2^ of 0.017 (on top of the 0.139 explained by the confounders) and the calculated power was at 0.61. According to the data the number of patients to achieve study power and significance level was set to a total of 392 patients.

### Subject selection

This single-center study enrolled all patients, who had a standardized admission procedure at the Saarland University Medical Center, Homburg, Germany and met the study inclusion criteria. Inclusion criteria were age > 18 years and prehospital intubation, catecholamine administration (intravenous epinephrine, norepinephrine and dobutamine at any dose or cafedrine/theodrenaline ≥ 200/10 mg) or fluid resuscitation ≥ 1.000 ml. Exclusion criteria were ongoing CPR when entering the hospital, secondary transport, and if either prehospital records or hospital protocols were missing. Patients were categorized based on whether they received prehospital IBP intervention or NIBP.

### Intervention

For the study period a material bag for arterial puncture and IBP measurement (puncture needles, disinfectants, compresses, adhesive dressings, sterile gloves and fenestrated drapes) was kept on two study vehicles. For monitoring we used an IBP module from GS Stemple corpuls^3^. The emergency physicians were anesthesiologists with at least 2 years of clinical experience. Establishment of IBP during prehospital treatments was at the discretion of treating emergency physicians. IBP line was placed on scene or in the ambulance before transportation in the radial (direct needle puncture or Seldinger technique) or femoral artery (Seldinger technique). As rescue option an ultrasound (Vscan Air, GE Healthcare) was permitted. The procedural instructions provided a maximum puncture time of 5 min or 2 puncture attempts.

### Measurements

Data were used from prehospital records, anesthesia protocols and emergency protocols from the resuscitation room. Diagnosis categories were taken from the prehospital records. The shock index was measured at two distinct time points. The initial shock index was taken from prehospital records, using the first documented values. The second shock index measurement was determined upon hospital admission, using the first documented value recorded after the patient’s arrival. Non-measurable low blood pressures were defined as systolic < 60 mmHg and for the regression analysis patients with cardiopulmonary resuscitation with a shock index of 3. Medical histories were taken from the in-hospital patient records. To calculate the total number of comorbidities and pre-existing conditions, we summed the following for all individuals: arterial hypertension, hyperlipidemia, smokers, known drug/alcohol abuse, diabetes, atrial fibrillation, cardiovascular disease, peripheral arterial disease, cerebrovascular disease, chronic lung disease, chronic liver disease, chronic kidney disease, hemodialysis, dementia, human immunodeficiency virus infection, and known tumor disease.

### Data analysis

Data were analyzed using IBM SPSS Statistics version 29.0.2.0. Power analysis was conducted with PASS 2022 Power Analysis and Sample Size Software version 22.0.3. Normality was assessed with the Kolmogorov–Smirnov test. Group comparisons for mean values and standard deviations were performed using the Student's t-test for parametric data and the Mann–Whitney U test for nonparametric data. Categorical variables were analyzed using the chi-square test or Fisher's exact test in case of an expected frequency n < 5. Multiple linear regression models with the forward selection method were used to assess shock index adjusting for univariate significant confounders. Confounders analyzed were age, study vehicle, prehospital intubation, prehospital CPR, trauma, initial blood pressure (BP) systolic(syst.) < 90 mmHg, first calculated shock index, initial Glasgow Coma Scale (GCS) and sum of comorbidities. A significance level of α < 0.05 was applied.

## Results

**Fig. 1:**
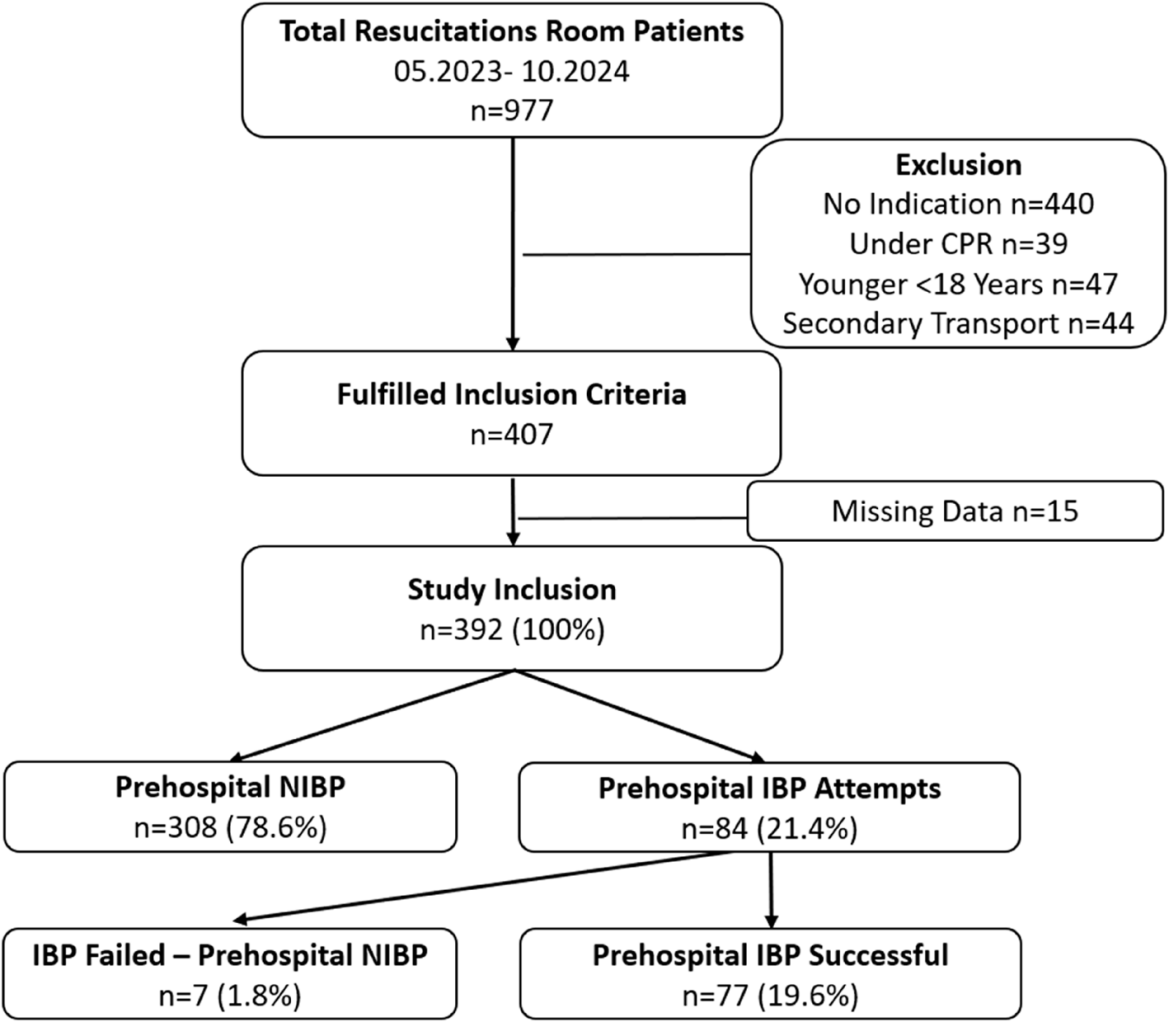
Flow diagram showing study inclusions criteria in relation to the total number of resuscitation room patients. IBP = invasive blood pressure, NIBP = noninvasive blood pressure

From May 2023 to November 2024 a total of 977 patients were admitted to the resuscitation room. Of n = 407 (41.7%) who fulfilled inclusion criteria n = 392 (40.1%) were included. Of the included patients a total of n = 84 (21.4%) had a prehospital IBP attempt, which succeeded in n = 77 (19.6%) (Fig. 1.).

Inclusion criteria were fulfilled by intubation (n = 276; 70.4%), catecholamine administration (n = 214, 54,6%) and/or fluid resuscitation (n = 202, 51,5%). 240 (61.2%) patients had more than one study inclusion criterion. Cardiovascular failure and cardiopulmonary resuscitation n = 157 (40%) were the most prevalent underlying conditions. Demographic and operational data and diagnosis categories are shown in Table 1. Sixteen patients (4.1%) presented with an unmeasurable low initial systolic blood pressure; for analysis, their SBP was imputed as 59 mmHg, yielding a mean shock index of 2.5 ± 1.0.

**Table 1:** Demographic and prehospital parameters of included patients. Prehospital time is the time from first contact until handover in the hospital. P-value for comparison categorical variables were analyzed using the chi-square test or Fisher's exact test in case of n < 5, respectively with p < 0.05. P-Value for comparison parametric data using an independent sample t-test and Mann–Whitney-U Test for nonparametric data with a p < 0.05. BP = blood pressure CAD = coronary artery disease, CKD = chronic kidney disease, CNS = central nervous system, CPR = cardiopulmonary resuscitation, GCS = Glasgow Coma Scale, ISS = Injury Severity Score, sys. = systolic, psychiatric = suspected suicide with overdose/intoxication

	**Total**	**NIBP**	**IBP**	**p-value**
Total (%)	392 (100%)^1^	315(80.4%)^1^	77(19.6%)^1^	
***Demography***				
Male(n)	258(65.8%)	209 (66.3%)	49 (63.6%)	0.69
Female(n)	134 (34.2%)	106 (33.7%)	28 (36.4%)	0.69
Age	64 ± 17	63 ± 17	67 ± 17	0.12
***Medical History***				
Hypertension(n)	196 (50%)	149 (47.3%)	47(61%)	0.04*
Cardiovascular (n)	172 (43.9%)	139 (44.1%)	33 (42.9%)	0.47
Diabetes (n)	101 (25,8%)	85 (27%)	16 (20.8%)	0.17
CAD (n)	77 (19.6%)	61 (19.4%)	16 (20.8%)	0.45
CKD (n)	45(11.5%)	36 (11.4%)	9 (11.7%)	0.54
Sum of all comorbidities (n)	2 ± 2	2 ± 2	2 ± 2	0.79
***Prehospital parameters:***				
First GCS (3–15)	8 ± 5	8 ± 5	8 ± 5	0.73
Initial HR (bpm)	104 ± 38	104 ± 39	102 ± 36	0.64
Initial Bp (syst. mmHg)	118 ± 46	118 ± 45	118 ± 49	0.96
Initial Shock Index	1.08 ± 0.7	1.08 ± 0.7	1.09 ± 0.7	0.9
Initial CPR (n)	91 (23.2%)	78 (24.8%)	13 (16.9%)	0.18
Initial Syst. BP (< 90 mmHg)	181 (46.2%)	146 (46.3%)	35 (45.5%)	0.9
Prehospital Intubation(n)	276 (70.4%)	224 (71.1%)	52 (67.5%)	0.58
Prehospital time (min)	63 ± 17	62 ± 22	64 ± 20	0.42
***Diagnosis category:***				
Trauma (n)	82 (20.9%)	72 (22.9%)	10 (13.0%)	0.06
ISS-Score (0–75)	27 ± 15	28 ± 15	25 ± 7	0.32
CNS (n)	60 (15.3%)	48 (15.2%)	12 (15.6%)	1.0
Cardiovascular (n)	157 (40.1%)	127 (40.3%)	30 (39%)	0.9
Pulmonary (n)	25 (6.4%)	20 (6.3%)	5 (6.5%)	1.0
Abdomen (n)	12 (3.1%)	7 (2.2%)	5 (6.5%)	0.07
Psychiatric (n)	15 (3.8%)	12 (3.8%)	3 (3.9%)	1.0
Others (n)	41 (10.4%)	29 (9.2%)	12 (15.6%)	0.14

^1^Data are shown as n (%) or mean (± SD). * = p < 0.05 significant difference between the two groups

### Primary Endpoint

**Fig. 2:**
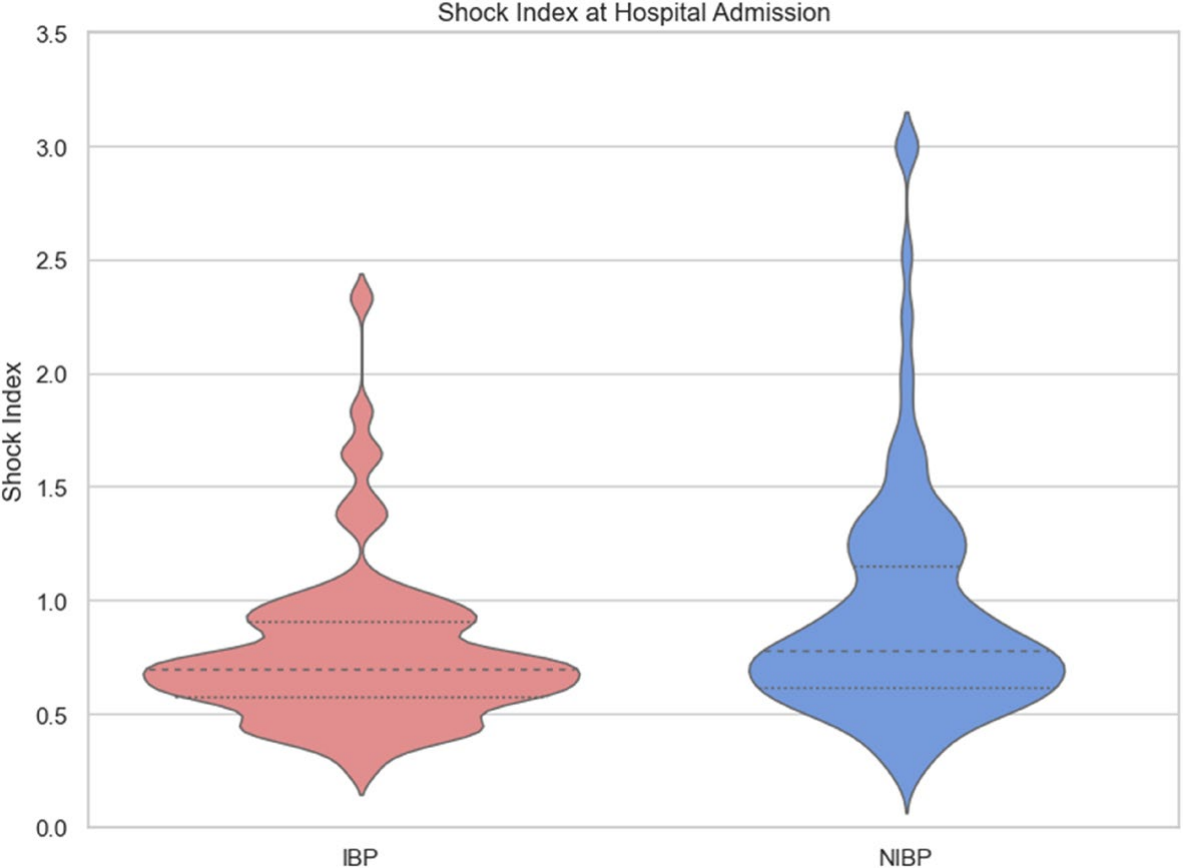
Violin plot of the Shock Index at hospital admission in comparison of IBP and NIBP. The dashed lines mark the median and the dotted line the interquartile range (bandwidth = 0.15)

Shock index at hospital admission was significantly lower in the IBP group compared with NIBP group (0.77 ± 0.4 vs. 0.93 ± 0.5; p = 0.002). After excluding the six patients who required starting CPR during handover, the difference remained significant (0.77 ± 0.40 vs. 0.89 ± 0.40; *p* = 0.011). Shock index > 1 at hospital admission was more frequent in the NIBP group (33% [n = 104]) than in the IBP group (15.6% [n = 12]; p = 0.003). Figure 2 shows the shock index at hospital admission.

In the univariate linear regression analysis, the following factors were significantly associated with the shock index at hospital admission: the initial shock index on scene (p < 0.001), initial BP sys. < 90 mmHg (p < 0.001), prehospital resuscitation (p < 0.001), IBP (p = 0.011), and trauma (p = 0.025).

**Fig. 3:**
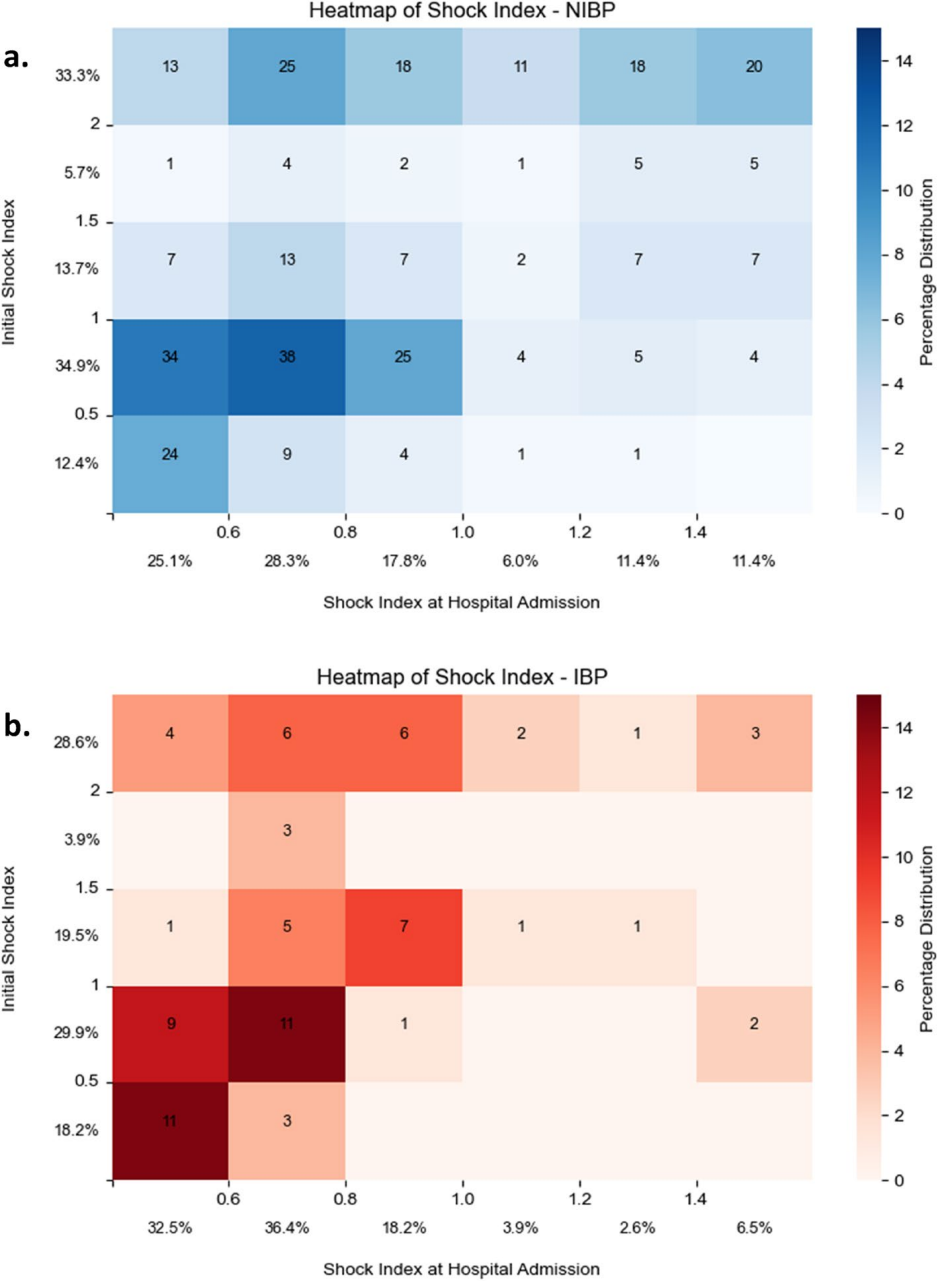
Heatmap showing the change of Shock Indices in n (%) from initial Shock Index on scene to Shock Index at hospital admission for a. NIBP (blue) and b. IBP (red). The comparison of the two Figures a and b indicates that IBP patients tended to have lower Shock Index at hospital admission, especially when their prehospital SI was lower than 2

The multiple linear regression analysis (n = 392) showed that IBP was independently associated with a significantly lower shock index at hospital admission by 0.154 (p = 0.008), after adjusting for all univariate significant factors (Table 2). Among patients with an indication for IBP, those who received IBP had lower shock index at admission compared with those who did not, even after adjusting for potential confounders. Since the initial shock index on scene had the greatest influence on the shock index at hospital admission, their relationship is illustrated in Fig. 3. Complete analysis of all confounders and IBP attempts in Supplement 2.

**Table 2:** Multiple regression analysis with significant predictors for the Shock index at hospital admission. Exclusions in the multiple regression analysis were trauma (p = 0.22) and BP sys. < 90 mmHg (p = 0.67).* = p < 0.05 significant factors within the regression analysis. CPR = cardiopulmonary resuscitation. CI = 95% Confidence Interval

Predictor	Regression Coefficient	Lower CI	Higher CI	p-value
Initial Shock Index on Scene Prehospital CPR IBP	+ 0.25 − 0.28 − 0.15	+ 0.18 − 0.44 − 0.27	+ 0.32 − 0.12 − 0.04	p < 0.001* p < 0.001* p = 0.008*

### Secondary endpoints

The average number of administered catecholamine boluses per patient was significantly higher in the IBP group (2.1 ± 2.5) compared with the NIBP group (1.2 ± 1.8 with p < 0.001). Push-dose vasopressors were given mainly as cafedrine/theodrenaline in 156 patients, while norepinephrine and epinephrine were used in 65 and 23 patients, respectively. Continuous catecholamine administration was also significantly more frequent in the IBP (35.1% [n = 27]) vs. NIBP-group (21.6% [n = 68]; p = 0.017). Furthermore, administering epinephrine and norepinephrine at doses above 0.1 µg/kg/min was more common in the IBP group (24.7% [n = 19] vs. 11.5% [n = 36]; p = 0.004).

There were no significant differences between groups regarding the administration of isotonic fluids, with an average of 974 ± 764 ml in the IBP group compared with 841 ± 606 ml in the NIBP group (p = 0.11), or in the use of hypertonic fluids in the IBP group (2.6% [n = 2]) vs. in the NIBP group (2.2% [n = 7]; p = 1.0).

Patients in the IBP group showed significantly higher pH values in the first arterial blood gas analysis (7.34 ± 0.13 vs. 7.25 ± 0.16; p < 0.001) and less negative base excess (− 3.8 ± 5.2 vs. − 6.0 ± 7.8; p = 0.004), while lactate levels were lower in the IBP group (3.6 ± 3.2 vs. 4.4 ± 4.2; p = 0.047).

### Exploratory analysis

The average prehospital time was 63 ± 17 min, with no significant difference between groups 62 ± 22 min in the NIBP vs. 64 ± 20 min in the IBP group (p = 0.42). No significant delay was observed even after analyzing for IBP attempts (Supplement 2- Intention-to-treat Analysis with IBP attempts).

A subgroup analysis based on the use of continuous catecholamine treatment via syringe pump is presented in Table 3.

**Table 3:** Subgroup of patients who received continuous catecholamine therapy. P-value for comparison categorical variables were analyzed using the chi-square test with p < 0.05. P-Value for comparison parametric data using an independent sample t-test with a p < 0.05. ^1^ mean(± SD) or n (%). * = p < 0.05 significant difference in the two groups. aBGA = first arterial blood gas analysis after admission, SI = Shock Index

	**Total**	**NIBP**	**IBP**	**p-value**
**Total numbers**	95^1^	68^1^	27^1^	
Epinephrine	7 (7.4%)	4 (5.9%)	3 (1.1%)	0.4
Norepinephrine	90 (94.7%)	65 (95.6%)	25 (92.6%)	0.62
Dobutamin	2 (2.1%)	2 (2.9%)	0	0.59
SI at hospital admission	0.98 ± 0.54	1.06 ± 0.6	0.76 ± 0.24	< 0.001*
SI > 1	36 (37.9%)	31 (45.6%)	5 (18.5%)	0.019*
pH in aBGA	7.21 ± 0.17	7.19 ± 0.18	7.27 ± 0.13	0.034*
Base Excess in aBGA	−8.4 ± 7.8	−9.1 ± 8.4	−6.7 ± 5.6	0.12
Lactate in aBGA	5.7 ± 4.7	5.8 ± 4.9	5.4 ± 3.9	0.7

Among the 392 study patients, 105 (26.8%) required prehospital cardiopulmonary resuscitation with 14 (18.2%) in the IBP group and 91 (28.9%) in the NIBP group. On admission, the shock index was significantly higher in patients who underwent CPR (1.04 ± 0.6) than in those who had not (0.89 ± 0.4; *p* = 0.004), with a mean difference of 0.19 (95% CI 0.06–0.31). After we excluded the six patients, who required starting CPR during handover, the mean difference between the two groups was no longer statistically significant (0.92 ± 0.4 vs. 0.89 ± 0.4; p = 0.158, 95% CI difference − 0.03 to 0.16). Table 4 shows the comparison of CPR patients between the IBP and NIBP groups.

**Table 4:** Subgroup of patients who received prehospital cardio-pulmonary resuscitation. P-value for comparison categorical variables were analyzed using the chi-square test with p < 0.05. P-Value for comparison parametric data using an independent sample t-test with a p < 0.05. ^1^ Data are presented as means (± SD) or n (%). * = p < 0.05 significant difference in the two groups. aBGA = first arterial blood gas analysis after admission, pVT = pulseless ventricular tachycardia, VF = ventricular fibrillation, PEA = Pulseless electrical activity, SI = Shock Index

	**Total**	**NIBP**	**IBP**	**p-value**
**Total numbers**	105	91	14	
**Initial CPR**	**91 (86.7%)**	**78 (85.7%)**	**13 (92.9%)**	**0.69**
*pVT/VF*	*44 (48.4%)*	*38 (48.7%)*	*6 (46.2%)*	
*Asystole/PEA*	*47 (51.6%)*	*40 (51.3%)*	*7 (53.8%)*	
**CPR during prehospital treatment**	**28 (26.7%)**	**26 (28.6%)**	**2 (14.3%)**	**0.34**
*pVT/VF*	*10 (35.7%)*	*9 (34.6%)*	*1 (50%)*	
*PEA*	*16 (57.1%)*	*15 (57.7%)*	*1 (50%)*	
Asystole	*2 (7.1%)*	*2 (7.7%)*	*0*	
**Parameters**				
Continuous catecholamine therapy	35 (33.3%)	27 (29.7%)	8 (57.1%)	0.06
SI > 1	40 (38.1%)	37 (40.7%)	3 (21.4%)	0.24
SI at hospital admission	1.04 ± 0.6	1.08 ± 0.6	0.75 ± 0.3	0.06
pH in aBGA	7.21 ± 0.17	7.13 ± 0.18	7.25 ± 0.14	0.02*
Base Excess in aBGA	−8.4 ± 7.8	−9.9 ± 8.7	−5.6 ± 5.6	0.08
Lactate in aBGA	5.7 ± 4.7	7.3 ± 4.5	4.7 ± 3.0	0.7

Pulseless electrical activity (PEA) was observed in 21 patients during the treatment and transport period, defined as the time from initial contact to 5 min after hospital admission. Of these, 20 patients were in the NIBP group (p = 0.058). The PEA occurred in 16 patients before hospital arrival and in 5 patients after admission, with four events being detected during the handover. All cases were nontrauma patients. The exact cause of PEA was unknown.

## Discussion

In this prospective nonrandomized interventional study, the use of prehospital IBP in critically ill patients was associated with an improved shock index at hospital admission. This finding remained statistically significantly even after adjusting for all tested confounders*.*

In the IBP group, the prevalence of a shock index > 1 was only half as frequent compared with patients without IBP even when the mean shock index was the same at first prehospital measurement. Hypertension was the only unevenly distributed variable between groups; however, since a shock index > 1 is associated with increased mortality even in patients treated with β-blockers [4], a relevant impact on the results is unlikely. The study demonstrates an association between prehospital monitoring using IBP and an improvement of a clinical outcome parameter.

Although the initial shock index strongly influenced the shock index at hospital admission, patients in the IBP group still showed more improved shock index upon arrival at hospital, suggesting that better monitoring resulted therapy may have contributed to a hemodynamic stabilization.

This improvement in the shock index can be attributed to several potential factors. The first indication for prehospital IBP in our study was the use of catecholamines. IBP allows for earlier detection of hypotension, enabling faster initiation of catecholamine therapy [5, 6]. IBP patients received catecholamines more frequently, both as bolus doses and as continuous infusions. It is likely that fewer patients in the NIBP group received catecholamines, despite being clinically indicated. This may explain why the NIBP group exhibited a significantly lower pH and base excess, as well as a higher lactate level indicating greater metabolic acidosis at hospital admission. With approximately the same volume administration, we assume that there was no dilution effect.

Furthermore, the administration of higher catecholamines via syringe pumps varied significantly between the groups, with higher administered dosage in the IBP group. Although higher catecholamine doses have been linked to an increased risk of acute kidney injury [14], there is no evidence to suggest an association between short term high catecholamine doses and higher mortality rates or worse neurological outcome [15–17].

The group of patients who receives continuous catecholamine therapy represents the most vulnerable patients, strongly dependent on continuous and accurate blood pressure monitoring as NIBP measurement is less precise in detecting hypotension, with a standard deviation of over 20 mmHg being common [8, 10, 11]. Interestingly, in the subgroup analysis of continuous catecholamine therapy the absence of a significant difference in blood gas values between the groups suggests that while IBP improves systemic hemodynamic parameters, it may not directly influence all markers of tissue-level hypoxia or anaerobic metabolism within the prehospital timeframe. Since the half-life of lactate clearance is already 15 min in healthy patients and longer in critically ill patients, clearance is not meaningful within the timeframe of prehospital care [18]. However, the higher pH and base excess values in the IBP group indicate that metabolic derangements were less severe, reversible, potentially due to earlier detection of hemodynamic instability and faster initiation of catecholamine therapy.

Prehospital intubation was the second indication for IBP placement in our study. Up to 22% of all patients with prehospital airway management become hypotensive with systolic blood pressure dropping below 90 mmHg [19, 20] and these patients have a significantly higher mortality rate [19]. However, in the NIBP group, blood pressure measurements were often performed at long and irregular intervals, and the accuracy of the values is limited [12]—particularly during critical phases such as intubation. As a result, the true extent and frequency of hypotensive episodes likely remain underestimated.

As specified in the study protocol, crews of all study vehicles were required to attempt placement of an IBP line in every patient who met the inclusion criteria. Whenever this mandate was not followed, the responsible physician had to submit a written explanation of the deviation. Reasons for deviations included lack of motivation, a short transport distance, and oversights in emergency. In the regression model the study vehicle had no effect on the shock index at hospital admission. A better medical care of patients within the study vehicle is therefore unlikely.

A common criticism of IBP is the potential for prolonged prehospital treatment times. While previous studies [7] reported no significant increase in on-scene times, other data suggest a delay in prehospital time [8]. While our data showed no significant difference in total prehospital time between groups after adjusting for IBP attempts, we did not account for actual transport duration. It is therefore possible that IBP placement slightly extends on-scene time. However, the potentially longer on-scene time may be compensated by a shorter duration in the resuscitation room, thereby balancing the overall time to definitive diagnosis and treatment [21, 22]. Moreover, this longer prehospital time is used to extend advanced hemodynamic monitoring, thereby reducing uncertain monitoring intervals.

During our study period, PEA was observed in 5.4% of all patients in the prehospital phase until 5 min after handover and PEA occurred more frequently in the NIBP group. It should be noted that 4 of these patients in the NIBP group presented with PEA during the handover situation at hospital admission. We assume that PEA occurs less often in the IBP group because IBP leads to a faster response diagnosis and in targeted catecholamine therapy [23].

Using the shock index as the primary endpoint is open to debate, as shock index has had little influence on clinical decision-making and is rarely evaluated as a primary outcome in prospective studies. While shock index offered an objective, readily obtainable surrogate for hemodynamic compromise in our study, future investigations should prioritize more patient centered outcomes. Composite metabolic targets (e.g., lactate or base-excess normalization), the incidence of prehospital PEA, or even mortality would provide more clinically meaningful evidence of the benefit of prehospital IBP.

We advocate the use of prehospital IBP measurement in selected patients, because we saw an association between IBP and hemodynamic stability. At present, the implementation of invasive blood pressure (IBP) monitoring appears to be feasible within physician-staffed EMS systems [9]. However, maintaining the necessary equipment and ensuring technical availability for IBP monitoring involves increased logistical effort and additional costs. Nevertheless, since vehicles may already serve dual purposes, including use for secondary intensive care transport, the required equipment is often available, thereby limiting additional costs to single-use items.

## Limitations

Our study has several limitations. We acknowledge that the exclusive inclusion of patients admitted to our own center may have led to a bias. The most important limitation is the absence of blinding and the lack of real-time data on vital signs. Since prehospital data on area under the curve and hypotension duration are missing, the causality of improvement cannot be confirmed with a high degree of certainty. Nearly half of the prehospital vital signs were taken from handwritten documentation and may not be accurate. Additionally, IBP always provides pressure reading, whereas NIBP is prone to significant measurement fluctuations, making direct comparability between the two methods difficult. Timing of catecholamine administration was not documented and may have been initiated before the establishment of IBP. Furthermore, prehospital blood pressure drops, and the duration of hypotension could not be measured accurately. As the study was conducted without randomization, the IBP group may have been subject to selection imbalances. The qualifications and experience of physicians in both groups were not recorded but could have influenced the results. Lastly, it remains unclear whether our findings translate into reduced morbidity or mortality.

## Conclusion

In conclusion, IBP offers significantly advantages in prehospital setting, particularly associated with a lower shock index on admission and may help enabling goal-directed therapy. IBP use was linked to more frequent continuous catecholamine administration and to higher administered doses. By detecting impending hemodynamic instability earlier than NIBP, IBP may enable early treatment and thereby help prevent deterioration into PEA. These benefits appear to outweigh the potential downsides, such as potential longer prehospital times. Based on this finding and the low IBP failure rates the authors suggest that IBP is recommended for suspected prehospital hemodynamically unstable patients. However, further studies including randomized controlled trials are needed to define the specific therapeutic impact of prehospital IBP in critically ill patients on morbidity and mortality.

## Supplementary Information

Supplementary Material 1

Supplementary Material 2

## Data Availability

The datasets analyzed during the current study are not publicly available but can be requested from the corresponding author upon reasonable request.

## Abbreviations

aBGA: Arterial blood gas analysis
BP: Blood pressure
CAD: Coronary artery disease
CKD: Chronic kidney disease
CNS: Central nervous system
CPR: Cardiopulmonary resuscitation
IBP: Invasive blood pressure
ISS: Injury severity score
MAP: Mean arterial pressure
NIBP: Non-invasive blood pressure
PEA: Pulseless electrical activity
RSI: Rapid sequence induction
Sys.: Systolic
