# Supplementary material for: Prehospital invasive vs. non-invasive blood pressure monitoring: Impact on shock index at hospital admission in critically ill patients – a prospective intervention study
Source: Scand J Trauma Resusc Emerg Med. 2025 Oct 15;33:167. doi: 10.1186/s13049-025-01467-3 (PMC12522479; doi:10.1186/s13049-025-01467-3)
Supplement: Supplementary file 2 — Supplementary Material 2 [file 13049_2025_1467_MOESM2_ESM.docx]

**Supplement:**

**IBP Regression Model:**

**Supplement Table 1. Univariable regression analysis of potential influencing factors on shock index at hospital admission. Study vehicles are emergency physician cares with available IBP. Calculation of total number of comorbidities and pre-existing conditions: arterial hypertension, hyperlipidemia, smokers, known drug/alcohol abuse, diabetes, atrial fibrillation, cardiovascular disease, peripheral arterial disease, cerebrovascular disease, chronic lung disease, chronic liver disease, chronic kidney disease, hemodialysis, dementia, human immunodeficiency virus infection, and known tumor disease.**

| **Variable** | **n** | **R** | **R²** | **Regression Coefficient B** | **Lower 95% CI** | **Lower 95% CI** | **p-value** |
| --- | --- | --- | --- | --- | --- | --- | --- |
| Age in years | 392 | .057 | .001 | .002 | -.001 | .004 | 0.257 |
| BP syst. < 90 | 392 | .27 | .073 | .262 | .169 | .356 | **<.001*** |
| First calculated SI | 392 | .34 | .116 | .159 | .116 | .203 | **<.001*** |
| IBP | 392 | .129 | 0.17 | -.157 | -.278 | -.036 | **.011*** |
| Initial GCS | 392 | .009 | -.002 | .001 | -.009 | .01 | .866 |
| Prehospital CPR | 392 | .167 | .028 | .182 | .075 | .289 | **<.001*** |
| Prehospital Intubation | 392 | .011 | .000 | -.012 | -.118 | .094 | .822 |
| Study vehicle | 392 | .07 | .005 | -.07 | -.17 | .029 | 0.166 |
| Sum of Pre-existing Conditions | 392 | .014 | .000 | -.003 | -.029 | .022 | .787 |
| Trauma | 392 | .113 | .013 | -.135 | -.253 | -.017 | **.025*** |

*= significant on a p<0.05; BP= blood pressure; CPR= cardiopulmonary resuscitation; GCS= Glascow-Coma-Scale; IBP= invasive blood pressure; syst.= systolic.

**Table 2. Multiple regression analysis with all univariate significant factors:**

| **Model** | **N** | **R** | **R²** |  | **Regression Coefficient B** | **Lower 95% CI** | **Lower 95% CI** | **p-value** |
| --- | --- | --- | --- | --- | --- | --- | --- | --- |
| 1 | 392 | .34 | .116 | Constant  First calculated SI | .654  .159 | .573  .116 | .734  .203 | **<.001***  **<.001*** |
| 2 | 392 | .372 | .139 | Constant  First calculated SI  Prehospital CPR | .593  .243  -.259 | .505  .177  -.418 | .681  .313  -.100 | **<.001***  **<.001***  **0.001*** |
| 3 | 392 | .393 | .154 | Constant  First calculated SI  Prehospital CPR  IBP | .624  .248  -.278  -.153 | .533  .181  -.436  -.265 | .714  .316  -.120  -.040 | **<.001***  **<.001***  **<.001***  **.008*** |

Model 1: Constant, First Measured Shock Index

Model 2: Constant, First Measured Shock Index, Prehospital CPR

Model 3: Constant, First Measured Shock Index, Prehospital CPR, IBP

*= significant on a p<0.05

CPR= cardiopulmonary resuscitation; IBP= invasive blood pressure.

**Table 3. Exclusions in the regression analysis**

| **Model** | **Excluded Variable** | **p-Value (Sig.)** |
| --- | --- | --- |
| 1 | IBP | 0,02 |
| 1 | Trauma | 0,607 |
| 1 | BP syst. < 90 | 0,895 |
| 1 | Prehospital CPR | 0,001 |
| 2 | IBP | 0,008 |
| 2 | Trauma | 0,42 |
| 2 | BP syst. < 90 | 0,548 |
| 3 | Trauma | 0,224 |
| 3 | BP syst. < 90 | 0,671 |

BP= blood pressure; CPR= cardiopulmonary resuscitation; IBP= invasive blood pressure; syst.= systolic.

**Intention to treat Analysis IBP-Attempts:**

**Table 4.** Demographic and operational data of included patients. P-value for comparison categorial variables were analyzed using the chi-square test or Fisher's exact test in case of n<5 with p<0.05. P-Value for comparison parametric data using an independent sample t-test and Mann-Whitney-U Test for no parametric data with a p<0.05.

|  | **Total** | **Non-IBP-attempt** | **IBP-attempt** | **P-value** |
| --- | --- | --- | --- | --- |
| Total (%) | 392 (100%)¹ | 308 (100%)¹ | 84 (100%)¹ |  |
| ***Demography***  Male(n)  Female(n)  Age | 258(65.8%)  134 (34.2%)  64 ± 17 | 204(66.2%)  104(33.8%)  63 ± 17 | 54(64.3%)  30(35.7%)  67 ± 17 | 0.74  0.74  0.03* |
| ***Medical History***  Hypertension(n)  Cerebro-Cardiovascular (n)  Diabetes Mellitus(n)  CAD(n)  CKD(n)  Sum of all comorbidities (n) | 196 (50%)  172 (43.9%)  101 (25,8%)  77 (19.6%)  45(11.5%)  2 ± 2 | 149 (48.5%)  135 (44.0%)  83 (27%)  58 (18.9%)  36 (11.7%)  2 ± 2 | 47 (55.3%)  37 (43.5%)  18 (21.2%)  19 (22.4%)  9 (10.6%)  2 ±2 | 0.22  0.97  0.31  0.44  0.80  0.69 |
| ***Operational data:***  First GCS (3-15)  Initial HR in bpm  Initial Bp in mmHg  Initial CPR (n)  Syst. BP <90 mmHg  Prehospital Intubation(n)  Prehospital time (min) | 8 ± 5  104 ± 38  118 ± 46  91 (23.2%)  181 (46.2%)  276 (70.4%)  63 ± 17 | 8 ± 5  104 ± 39  118 ± 49  75 (24.3%)  141 (45.8%)  218 (70.8%)  62 ± 22 | 8 ± 5  102 ± 36  118 ± 45  16 (19.4%)  40 (47.6%)  58 (69%)  65 ± 20 | 0.17  0.65  0.96  0.31  0.76  0.76  0.24 |
| ***Diagnosis category:***  Trauma (n)  ISS-Score (0-75)  CNS (n)  Cardiovascular (n)  Pulmonary (n)  Abdomen (n)  Psychiatric (n)  Others (n) | 82 (20.9%)  27 ± 15  60 (15.3%)  157 (40.1%)  25 (6.4%)  12 (3.1%)  15 (3.8%)  41 (10.4%) | 72 (23.4%)  28 ± 16  48 (15.6%)  121 (39.3%)  20 (6.5%)  7 (2.3 %)  12 (3.9%)  28 (9.1%) | 10 (11.9%)  25 ± 7  12 (14.3%)  36 (42.9%)  5 (6.0%)  5 (6.0%)  3 (3.6%)  13 (15.5%) | 0.02*  0.32  0.77  0.55  0.86  0.08  0.89  0.09 |

¹Data are shown as n (%) or mean (±SD). * = p<0.05 significant difference in the two groups.

BP= blood pressure CAD= coronary arterial disease, CKD= chronic kidney disease, CNS =central nervous system, CPR= cardiopulmonary resuscitation, GCS= Glascow coma scale, IBP= invasive blood pressure; ISS= Injury Severity Score, syst.= systolic.

**Intention to treat Analysis IBP-Attempts Regression Model:**

**Supplement Table 5. Univariable regression analysis of potential influencing factors on shock index at hospital admission.**

| **Variable** | **n** | **R** | **R²** | **Regression Coefficient B** | **Lower 95% CI** | **Lower 95% CI** | **p-value** |
| --- | --- | --- | --- | --- | --- | --- | --- |
| Age in years | 392 | .057 | .001 | .002 | -.001 | .004 | 0.257 |
| BP syst. < 90 | 392 | .27 | .073 | .262 | .169 | .356 | **<.001*** |
| First calculated SI | 392 | .34 | .116 | .159 | .116 | .203 | **<.001*** |
| IBP-Attempts | 392 | .132 | 0.17 | -.156 | -.273 | -.039 | **0.009*** |
| Initial GCS | 392 | .009 | -.002 | .001 | -.009 | .01 | .866 |
| Prehospital CPR | 392 | .167 | .028 | .182 | .075 | .289 | **<.001*** |
| Prehospital Intubation | 392 | .011 | .000 | -.012 | -.118 | .094 | .822 |
| Study vehicle | 392 | .07 | .005 | -.07 | -.17 | .029 | 0.166 |
| Sum of Pre-existing Conditions | 392 | .014 | .000 | -.003 | -.029 | .022 | .787 |
| Trauma | 392 | .113 | .013 | -.135 | -.253 | -.017 | **.025*** |

*= significant on a p<0.05; BP= blood pressure; CPR= cardiopulmonary resuscitation; GCS= Glascow-Coma-Scale; IBP= invasive blood pressure; syst.= systolic.

**Table 6. Multiple regression analysis with all univariate significant factors:**

| **Model** | **N** | **R** | **R²** |  | **Regression Coefficient B** | **Lower 95% CI** | **Lower 95% CI** | **p-value** |
| --- | --- | --- | --- | --- | --- | --- | --- | --- |
| 1 | 392 | .34 | .116 | Constant  First calculated SI | .654  .159 | .573  .116 | .734  .203 | **<.001***  **<.001*** |
| 2 | 392 | .372 | .139 | Constant  First calculated SI  Prehospital CPR | .593  .243  -.259 | .505  .177  -.418 | .681  .313  -.100 | **<.001***  **<.001***  **0.001*** |
| 3 | 392 | .395 | .156 | Constant  First calculated SI  Prehospital CPR  IBP-attempts | .626  .249  -.277  -.157 | .536  .181  -.435  -.266 | .716  .317  -.119  -.048 | **<.001***  **<.001***  **<.001***  **.005*** |

Model 1: Constant, First Measured Shock Index

Model 2: Constant, First Measured Shock Index, Prehospital CPR

Model 3: Constant, First Measured Shock Index, Prehospital CPR, IBP

*= significant on a p<0.05

CPR= cardiopulmonary resuscitation; IBP= invasive blood pressure.

**Table 7. Exclusions in the regression analysis**

| **Model** | **Excluded Variable** | **p-Value (Sig.)** |
| --- | --- | --- |
| 1 | IBP-attempts | 0,012 |
| 1 | Trauma | 0,607 |
| 1 | BP syst. < 90 | 0,895 |
| 1 | Prehospital CPR | 0,001 |
| 2 | IBP-attempts | 0,005 |
| 2 | Trauma | 0,42 |
| 2 | BP syst. < 90 | 0,548 |
| 3 | Trauma | 0,224 |
| 3 | BP syst. < 90 | 0,671 |

BP= blood pressure; CPR= cardiopulmonary resuscitation; IBP= invasive blood pressure; syst.= systolic.
